# Serpinb1a Is Dispensable for the Development and Cytokine Response of Invariant Natural Killer T Cell Subsets

**DOI:** 10.3389/fimmu.2020.562587

**Published:** 2020-11-11

**Authors:** Nathan G. F. Leborgne, Adriano Taddeo, Stefan Freigang, Charaf Benarafa

**Affiliations:** ^1^ Institute of Virology and Immunology, Mittelhäusern, Switzerland; ^2^ Department of Infectious Diseases and Pathobiology, Vetsuisse Faculty, University of Bern, Bern, Switzerland; ^3^ Graduate School for Cellular and Biomedical Sciences, University of Bern, Bern, Switzerland; ^4^ Institute of Pathology, University of Bern, Bern, Switzerland

**Keywords:** invariant NKT, serpin, α-galactosylceramide, cytokine response, innate immunity

## Abstract

Invariant natural killer T (iNKT) cells are innate-like T lymphocytes. They quickly respond to antigenic stimulation by producing copious amounts of cytokines and chemokines. iNKT precursors differentiate into three subsets iNKT1, iNKT2, and iNKT17 with specific cytokine production signatures. While key transcription factors drive subset differentiation, factors that regulate iNKT subset homeostasis remain incompletely defined. Transcriptomic analyses of thymic iNKT subsets indicate that *Serpinb1a* is one of the most specific transcripts for iNKT17 cells suggesting that iNKT cell maintenance and function may be regulated by *Serpinb1a.* Serpinb1a is a major survival factor in neutrophils and prevents cell death in a cell-autonomous manner. It also controls inflammation in models of bacterial and viral infection as well as in LPS-driven inflammation. Here, we examined the iNKT subsets in neutropenic *Serpinb1a* ^−/−^ mice as well as in *Serpinb1a* ^−/−^ mice with normal neutrophil counts due to transgenic re-expression of SERPINB1 in neutrophils. In steady state, we found no significant effect of *Serpinb1a*-deficiency on the proliferation and numbers of iNKT subsets in thymus, lymph nodes, lung, liver and spleen. Following systemic activation with α-galactosylceramide, the prototypic glycolipid agonist of iNKT cells, we observed similar serum levels of IFN-γ and IL-4 between genotypes. Moreover, splenic dendritic cells showed normal upregulation of maturation markers following iNKT cell activation with α-galactosylceramide. Finally, lung instillation of α-galactosylceramide induced a similar recruitment of neutrophils and production of iNKT-derived cytokines IL-17, IFN-γ, and IL-4 in wild-type and *Serpinb1a* ^−/−^ mice. Taken together, our results indicate that Serpinb1a, while dominantly expressed in iNKT17 cells, is not essential for iNKT cell homeostasis, subset differentiation and cytokine release.

## Introduction

Invariant natural killer T (iNKT) cells are tissue resident innate-like T lymphocytes that respond to lipid antigens. Upon T cell receptor (TCR) activation, they promptly release cytokines that modulate inflammation and adaptive responses. In addition, iNKT cells contribute to tissue homeostasis in the thymus medulla and in the periphery (1). The TCR of iNKT cells is specific for exogenous and endogenous lipid antigens presented by CD1d, a non-polymorphic MHC class I-like molecule. This specificity is mediated by their semi-invariant TCR composed of an invariant TCRα chain (V_α_14-J_α_18 in mice, V_α_24-J_α_18 in humans) paired with a limited set of TCRβ chains (V_β_2, V_β_7, or V_β_8 in mice, V_β_11 in humans) (2). iNKT cell development in the thymus initially overlaps with that of conventional T cells until the double negative (CD4^neg^CD8^neg^) stage 4 (DN4). However, iNKT cells differ from conventional T cells by expressing the transcription factor PLZF, which is common to innate-like T cells (3, 4). The majority of iNKT cells proceed to the double positive stage (DP, CD4^+^CD8^+^) and they are selected following strong interactions with other DP thymocytes expressing CD1d in the thymus medulla (5–7). Yet, one subset of iNKT cells differentiates already at the double negative (DN) stage and remains CD4^neg^ (8).

iNKT cells migrate from the thymus to peripheral organs, where they are found both as DN as well as CD4^+^CD8^neg^ in mouse peripheral organs. As mostly tissue resident cells, their localization within different organs influences cytokine production and pathophysiological processes (9). Mature iNKT cells are classified into functionally heterogeneous subsets described as iNKT1, iNKT2, and iNKT17, named by analogy to T helper cells. Differential expression levels of PLZF and of subset-specific transcription factors distinguish the iNKT1 expressing T-bet and low levels of PLZF; the iNKT2 expressing GATA-3 and high level of PLZF; and iNKT17 expressing ROR-γt and intermediate levels of PLZF (10–12). The signature cytokines of each of the three subsets are IFN-γ, IL-4, and IL-17, respectively (13, 14). Peptide MHC-dependent αβ T helper cells also produce these cytokines but require much more time for activation, proliferation and differentiation. iNKT cells in contrast can immediately produce large amounts of these key cytokines, which potentiate innate immunity and guide adaptive responses (13). iNKT cell expression of IL-4 and IL-13 appears essential in mouse models of airway inflammation and hyper-reactivity and there is some evidence that iNKT cells contribute to the pathogenesis of human asthma (15). IL-4 produced by iNKT cells was shown on the contrary to help resolution of sterile injury in the liver (16) and iNKT cells help reduce tumor growth and angiogenesis in different types of cancer by releasing INF-γ (17, 18). In models of *Streptococcus pneumoniae* lung infection, iNKT cells enhance bacterial clearance by producing IFN-γ and IL-17 and inducing the essential recruitment of neutrophils (19, 20). On the other hand, IL-17 produced by iNKT cells drives the inflammation in the joints of spondyloarthritis patients (21). A better understanding of molecular mechanisms that help fine tuning differentiation and cytokine production by iNKT cells is needed. The iNKT17 subset, in particular, is not as well defined as the iNKT1 and iNKT2 (22).

Transcriptomic studies revealed that *Serpinb1a* gene is highly and specifically expressed in the iNKT17 subset of both C57BL/6 and BALB/c mouse strains (23, 24). Moreover, the percentage of iNKT17 cells defined as CD138^+^ was increased in thymus and lung of *Serpinb1a^−/−^* (*Sb1a^−/−^*) mice (24). Whether Serpinb1a contributes to iNKT cell development, differentiation and function was not explored. Serpinb1a belongs to the serpin superfamily of protease inhibitors. Serpinb1a, as its human ortholog SERPINB1, is expressed in many leukocytes and is particularly abundant in the neutrophil cytoplasm. It is one of the best inhibitors of the main neutrophil serine proteases, cathepsin G, neutrophil elastase, and proteinase 3. Serpinb1a is a survival factor in neutrophils and monocytes by inhibiting cathepsin G-mediated cell-autonomous death. *Sb1a^−/−^* mice also produce more inflammatory cytokines in response to infection and endotoxin challenge. We thus explored here an essential function for Serpinb1 in iNKT cell subset differentiation and cytokine production.

## Methods

### Mice

C57BL/6J (WT) breeder mice were from the Janvier Laboratories (France). *Serpinb1a^tm1.1Cben^* (*Sb1a* ^−/−^) mice generated in 129S6/SvEvTac (129S6) and backcrossed in C57BL/6J background were described previously (25, 26). Transgenic mice expressing human SERPINB1 in neutrophils B6J-Tg(S100A8-hSERPINB1)3Cben (denoted SB1^PMN.Tg^) were crossed with *Sb1a* ^−/−^ as described previously (27). All experimental mice were therefore in the same C57BL/6J and produced in the SPF mouse unit of the Institute of Virology and Immunology. Mice were used between 8 and 25 weeks and were age- and sex-matched between genotypes for each experiment. Animal experimentation was conducted in compliance with the Swiss Animal Welfare legislation and animal studies were reviewed and approved by the commission on animal experiments of the canton of Bern, Switzerland under licenses BE8/16 and BE35/19.

### Blood and Tissue Leukocyte Isolation

Mice were euthanized and blood was collected in EDTA. Thymus, lungs, spleen, liver, and lymph nodes (inguinal, brachial, and axillary) were collected in cold PBS without cations supplemented with 1% FBS. Single cell suspensions of lymphoid organs and liver were obtained by successively passing diced tissue through 70- and 40-µm cell strainers. Liver cell suspensions were layered over 3 ml of lympholyte M solution and centrifuged at 1,000 g for 20 min at room temperature. Red blood cell lysis was performed on spleen and liver cells using ammonium chloride. Bronchoalveolar lavage fluid (BAL) was collected by three successive instillation of 1-ml PBS. Supernatant of the first BAL was used for cytokine quantification. The pellet was combined with cells of the other two BAL and processed for flow cytometry analysis. Cells were counted in a hemocytometer using Türk’s solution.

### Flow Cytometry and Cytokine Measurements

BV421-labelled CD1d tetramers preloaded with PBS57 (PBS57-tet) and unloaded CD1d tetramers were obtained from the National Institutes of Health tetramer core facility. Unloaded control tetramer was used as a negative control for each experiment. Fluorescently-labelled antibodies against mouse CD4 (clone RM4-5), CD122 (clone TM-β1), Ki-67 (clone 16A8), T-bet (clone 4B10), and PLZF (clone 9E.12) were from Biolegend and against CD3 (clone 145-2C11), TCRβ (clone H57-597), and RORγt (clone Q31-378) were from BD Biosciences. Dead cells were excluded with 7-AAD or LIVE/DEAD ﬁxable Aqua Dead stain kit (ThermoFisher Scientiﬁc, L34957). Blocking was done with CD16/CD32 (clone 2.4G2, Biolegend) prior to staining with cell surface antibodies. When required, cells were subsequently fixed and permeabilized using the Foxp3 staining buffer (BD biosciences) and incubated for detection of transcription factors and or proliferation (Ki-67, EdU). Acquisition of data was performed on a FACS-Canto II (BD Biosciences) and analyzed with FlowJo software version 10. Cytokines in BAL, cell supernatants and serum were measured by ELISA against IFN-γ, IL-4 (R&D Systems), and IL-17A (Invitrogen).

### *In Vivo* and *In Vitro* Models of Inflammation

Stock solutions of α-galactosyceramide (αGalCer) (Sigma, 67576) were prepared in DMSO at 1 mg/ml and stored at −20°C. For systemic activation, stock αGalCer was pre-diluted at 0.2 mg/ml in PBS containing 0.02% Tween 20 and further diluted 1:10 in PBS. Mice were injected intravenously in the tail vein with 2 µg αGalCer in 100 µl. For local lung inflammation, stock αGalCer was pre-diluted at 0.5 mg/ml in PBS with 0.02% Tween 20 and further diluted 1:5 in PBS. Mice were anesthetized with ketamine and xylazine (100 mg/kg and 10mg/kg, respectively) and intranasally instilled with 2µg αGalCer in 20 µl. In some experiments, the thymosine analog EdU (5-ethynyl-2’-deoxyuridine) was injected intravenously (160 µg in 100 µl/mouse) at the same time as αGalCer stimulation. Total lung cells were isolated using the mouse Lung Dissociation kit (Milneyi Biotec, 130-095-927) and proliferating (EdU^+^) iNKT cells were detected by flow cytometry using Click-iT EdU Cell Proliferation Kit (ThermoFisher Scientific, C10337). *In vitro* cytokine production was measured in the supernatant of isolated splenocytes (25 × 10^6^cells/ml) following a 48-h stimulation with indicated concentrations of αGalCer.

### Statistical Analysis

Statistical analysis was performed using Prism 8.0 (GraphPad, San Diego, CA). Mann-Whitney or one-way ANOVA were used to compare data from two or three genotypes, respectively. P < 0.05 was considered statistically significant.

## Results

**Figure 1:**
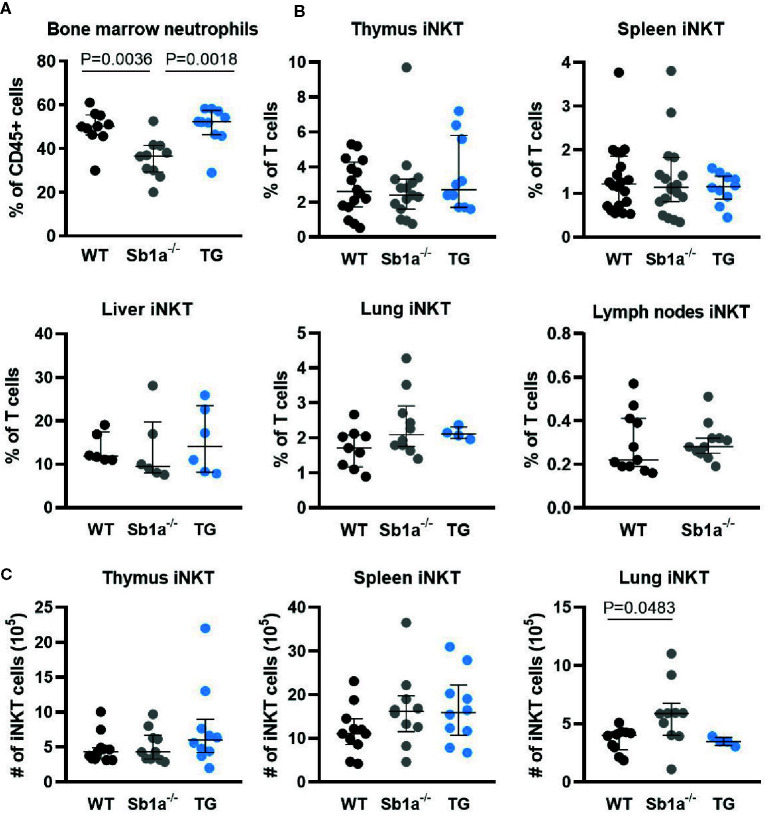
Ablation of *Serpinb1a* does not alter iNKT numbers in thymus and periphery. **(A)** Analysis of bone marrow neutrophils (Ly6G^+^). **(B)** Percentages of iNKT cells (CD1d-PBS57^+^, TCRβ/CD3^+^) in the thymus, spleen, liver, lung and lymph nodes. **(C)** Absolute numbers of iNKT cells in the thymus, spleen and lung. Data were from groups of 6- to 25-week-old mice matched for sex and age between genotypes. Data are from at least five independent experiments. Values for individual mice are shown with median and interquartile range.

To investigate whether Serpinb1a contributes to iNKT cell development and homeostasis, we analyzed iNKT cell subsets in WT, *Sb1a^−/−^* and *Sb1a^−/−^.SB1^PMNTg^* (TG) mice. The latter are constitutively deficient in endogenous mouse *Serpinb1a* and express human SERPINB1 in neutrophils, which rescues the neutropenic phenotype of *Sb1a^−/−^* mice (**Figure 1A**), as described previously (27). Percentage and absolute numbers of iNKT cells (PBS57-tet^+^) were similar in thymus and spleen of the different genotypes (**Figures 1B, C**). In contrast, we found an increase in absolute numbers of lung iNKT cells of *Sb1a^−/−^* mice, however the percentage of iNKT cells relative to T cells was comparable between wild-type and *Sb1a^−/−^* mice (**Figures 1B, C**). Percentage of iNKT cells in liver and lymph node iNKT cells were also similar in all genotypes (**Figure 1C**).

**Figure 2:**
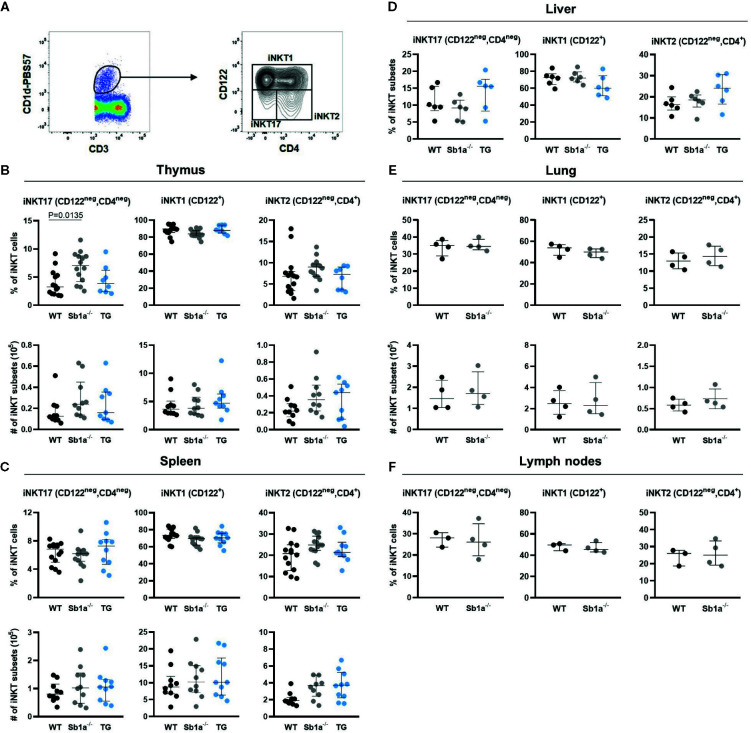
Surface marker expression analysis of iNKT subsets shows increased thymic iNKT17 cells in *Sb1a^−/−^* mice. **(A)** Representative flow cytometry plots of iNKT cell subsets based on CD122 and CD4 expression on CD1d-tet^+^ iNKT cells. Percentages and numbers of iNKT17, iNKT1, and iNKT2 in thymus **(B)**, spleen **(C)**, liver **(D)**, lung **(E)**, and lymph nodes **(F)** of mice of the indicated genotypes. Data were from groups of 6-25 week-old mice matched for sex and age between genotypes. Data are from at least four independent experiments. Values for individual mice are shown with median and interquartile range.

Analysis of iNKT subsets were first identified by differential expression of the surface markers CD4 and CD122 (**Figure 2A**). The percentage—but not absolute numbers—of iNKT17 (CD4^neg^CD122^neg^) cells were significantly increased in the thymus of *Sb1a^−/−^* mice compared to WT (P = 0.01) but not compared to *Sb1a^−/−^.SB1^PMNTg^* mice (P = 0.1) (**Figure 2B**). The numbers and percentages of iNKT17 (CD4^neg^CD122^neg^) cells in spleen, liver, lungs, and lymph nodes of *Sb1a^−/−^* mice were normal (**Figures 2C–F**). No difference between genotypes was observed for iNKT1 and iNKT2 numbers and proportions in all the tissues (**Figure 2**).

**Figure 3:**
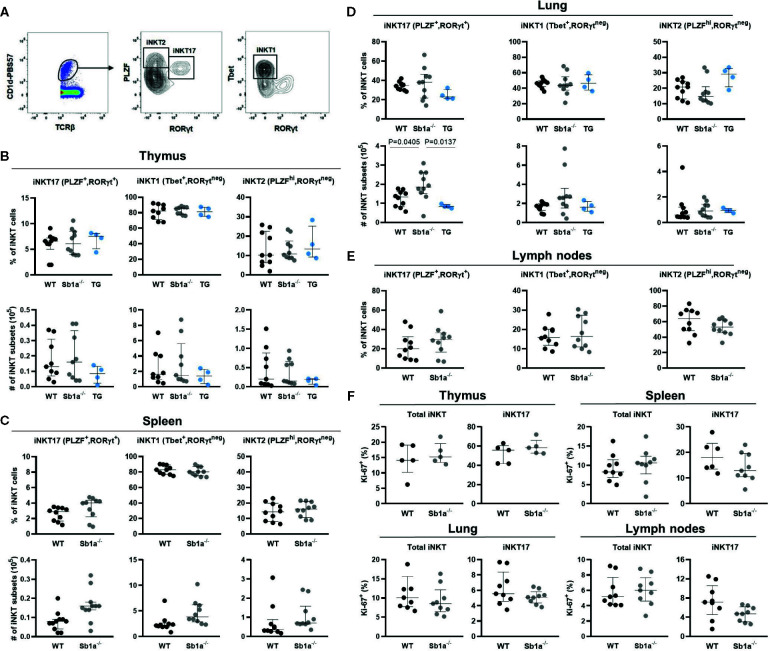
Transcription factor expression analysis of iNKT subsets indicates normal thymic iNKT17 cells in *Sb1a^−/−^* mice. **(A)** Representative flow cytometry plots of iNKT cell subsets based on PLZF, T-bet and RORγt expression on CD1d-tet^+^ iNKT cells. **(B)** Percentages and numbers of iNKT17, 1 and 2 in thymus **(B)**, spleen **(C)**, lung **(D)**, and lymph nodes **(E)**. Percentages of total iNKT and iNKT17 Ki-67^+^ cells in indicated organs. **(F)** Data were from groups of 6- to 8-week-old mice matched for sex and age between genotypes. Data are from at least four independent experiments. Values for individual mice are shown with median and interquartile range.

Because the delineation of the subsets is ambiguous using cell surface expression of CD4 and CD122 (**Figure 2A**), we further examined iNKT cell subsets in thymus, spleen lung, and lymph nodes using expression of the nuclear factors PLZF, T-bet, and RORγt, which provide a particularly clear identification of the iNKT17 cells (**Figure 3A**) (28). Using this analysis, percentages of iNKT17 cells were found to be identical in all genotypes in the different organs. Absolute numbers of iNKT17 cells but not other subsets were increased in lungs of *Sb1a^−/−^* mice and were similar in all other tissue (**Figures 3B–E**). We evaluated iNKT cell basal proliferation in the various organs by expression of Ki-67 at steady state. We found no difference between genotypes in Ki-67^+^ cells in total iNKT nor in the iNKT17 subset in any organ (**Figure 3F**). Overall, these findings do not support evidence for an essential function of Serpinb1a in iNKT17 cell subset homeostasis in steady state.

**Figure 4:**
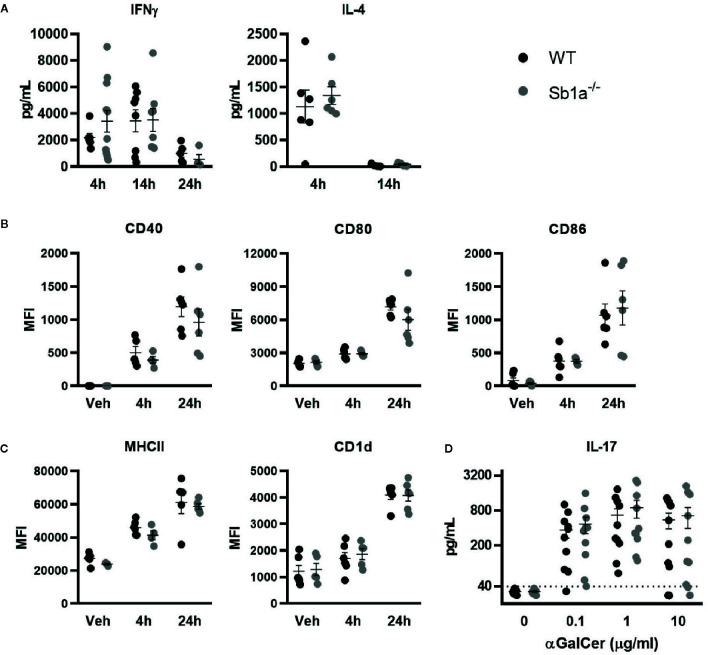
Normal cytokine release following systemic or *in vitro* activation of iNKT cells by αGalCer in *Sb1a^−/−^* mice. **(A–C)** WT and *Sb1a^−/−^* mice (6–8 weeks old) were injected intravenously with αGalCer. **(A)** Serum cytokine levels were measured by ELISA at indicated time points. **(B)** Dendritic cell expression of co-stimulatory molecules CD40, CD80, and CD86 and **(C)** antigen presenting molecules CD1d and MHCII were measured by flow cytometry (MFI, mean fluorescence intensity). Data from three independent experiments; n = 6–12/genotype. **(D)** Analysis of WT and *Sb1a^−/−^* splenocytes stimulated with indicated concentrations of αGalCer. IL-17, IFNγ, and IL-4 levels were measured by ELISA in the supernatant after 48 h. Data were from groups of 6- to 8-week-old mice matched for sex and age between genotypes. Data are from four independent experiments. Values for individual mice are shown as mean and SEM.

We then investigated iNKT activation *in vivo* following intravenous injection of α-galactosylceramide (αGalCer), the prototypic glycolipid activator of iNKT cells. αGalCer induced high levels of IFNγ and IL-4 in serum of WT and *Sb1a* ^−/−^ mice (**Figure 4A**). In contrast, induction of circulating IL-17A was not detected. We also observed that CD11c^+^ dendritic cells were similarly activated in the spleen of WT and *Sb1a* ^−/−^ mice at 4h and 24 h post-injection of αGalCer as indicated by increased expression of CD40, CD80, and CD86 (**Figure 4B**). Cell surface expression of CD1d and MHC-II were also augmented in a comparable manner in both genotypes at 4 and 24 h (**Figure 4C**). Therefore, *Serpinb1a* does not alter the strength of the response of iNKT cells or their ability to induce dendritic cell maturation in the spleen following systemic activation by αGalCer. *In vitro*, splenocytes of WT and *Sb1a* ^−/−^ mice were stimulated with increasing concentrations of αGalCer and cytokines were measured in supernatant after 48 h. A similar dose-dependent induction of IL-17A was measured in splenocytes of both genotypes (**Figure 4D**).

**Figure 5:**
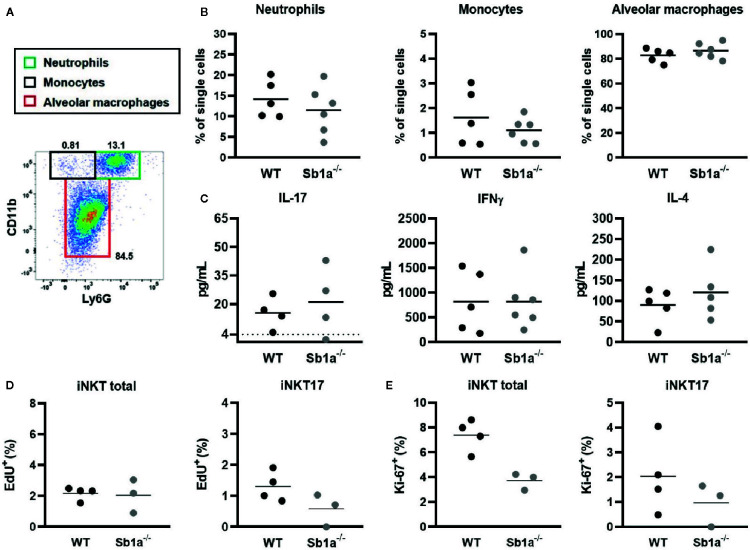
Lung instillation of αGalCer induces normal cytokine secretion and recruitment of inflammatory cells in *Sb1a^−/−^* mice. WT and *Sb1a^−/−^* mice were injected intranasally with 2μg αGalCer. **(A)** Representative flow cytometry plot of BAL cells 24 h after αGalCer instillation. **(B)** Neutrophil, monocyte and alveolar macrophage percentages in the BAL. **(C)** IL-17, IFNγ, and IL-4 BAL levels in BAL were measured by ELISA. Percentages of iNKT total and iNKT17 EdU^+^ cells **(D)** and Ki-67^+^ cells **(E)** in the lungs were measured by flow cytometry. Data were from groups of 6- to 12-week-old mice matched for sex and age between genotypes. **(A–C)** Data are from three independent experiments. Values for individual mice are shown with mean.

iNKT cells are present in the lung parenchyma and local instillation of αGalCer in the airways has been shown to induce neutrophil recruitment *via* IL-17 produced by NKT cells (29, 30). It was further shown that the RORγt^+^ iNKT17 subset was the specific source of the cytokine (28). To investigate the function of Serpinb1a in a lung model of local inflammation, we measured neutrophil recruitment, cytokine expression in BAL and iNKT proliferation 24 h after intranasal instillation of αGalCer (**Figure 5A**). We observed a similar recruitment of neutrophils and monocytes in the BAL of WT and *Sb1a* ^−/−^ mice (**Figure 5B**). Furthermore, expression of IL-17, IFNγ, and IL-4 in BAL were identical between genotypes (**Figure 5C**). Finally, total iNKT and iNKT17 proliferation in lungs (EdU^+^ and Ki-67^+^ cells) were equivalent in WT and *Sb1a^−/−^* mice (**Figures 5D, E**). Taken together, our results indicate that Serpinb1a does not significantly modify the inflammatory reaction induced by activation of lung iNKT cells.

## Discussion

Two independent transcriptomic studies have identified *Serpinb1a* as a gene highly, and specifically expressed in thymic iNKT17 cells using different cell surface marker strategies (23, 24). Here, we found that the percentages of iNKT17 cells defined as CD4^neg^CD122^neg^ were increased in thymus of *Sb1a* ^−/−^ mice. This finding was reminiscent of the data of Georgiev and colleagues showing that the percentage of CD138^+^ (iNKT17) cells was increased in thymus and lung of *Sb1a* ^−/−^ mice (24). However, a clear identification of iNKT subsets using cell surface markers remains challenging (31, 32) and is somewhat subjective because of the non-binary expression of the markers (**Figure 2A**). When we discriminated iNKT subsets using differential expression of transcription factors, we found no difference in thymus RORγt^+^ iNKT17 cells between genotypes. Furthermore, proportions of iNKT subsets in spleen, liver and lymph nodes were normal in *Sb1a* ^−/−^ mice using differential expression of CD4/CD122 or of transcription factors. In the lungs, absolute numbers – but not percentages – of total iNKT and of iNKT17 cells were increased in *Sb1a* ^−/−^ mice, a reflection of slightly increased total cell numbers. If this effect is biologically relevant, the lack of increased iNKT and iNKT17 cells in *Sb1a^−/−^.SB1^PMNTg^* mice indicate that the effect would not be iNKT cell-intrinsic and likely dependent on neutropenia. Proliferation of iNKT and iNKT17 cells was also similar in both genotypes. Taken together, these data indicate that deletion of *Serpinb1a* does not specifically promote RORγt^+^ iNKT17 subset differentiation, survival or migration relative to other subsets in steady state conditions. On the contrary, it is possible that Serpinb1a expression is regulated by RORγt. This hypothesis is supported by the transcriptomic studies of Mucosal Associated T (MAIT) cells that identified Serpinb1a as a marker for the MAIT17 subset defined by high expression levels of RORγt (33, 34). Overall, Serpinb1a expression in innate T cells is upregulated with or by RORγt and is associated with cells producing IL-17.

Serpinb1a is a survival factor for neutrophils and a regulator of inflammation. *Sb1a^−/−^* mice are neutropenic despite expressing 4-fold higher basal levels of G-CSF in blood (35). They consequently fail to clear *Pseudomonas aeruginosa* infection and present severe lung infection with high levels of TNFα and IL-1β (26). *Sb1a^−/−^* mice develop a more pronounced and protracted inflammation following influenza A virus infection with elevated levels of TNFα, IL-6, and IL-17 in the lungs (36). Similarly, systemic inflammation induced by LPS also causes increased cytokine production in *Sb1a^−/−^* mice (37, 38). Here, we found that local instillation of αGalCer in the lungs yielded normal production and of IL-4, IFNγ and IL-17 in BAL. These data suggest again that the modest increase iNKT and iNKT17 cells in the lungs of *Sb1a^−/−^* mice had no functional impact on IL-17 production. Similarly, splenocyte cultures pulsed with αGalCer also showed a normal IL-17 release. Finally, spleen dendritic cells were similarly activated in response to systemic injection of αGalCer in *Sb1a^−/−^* and WT mice. Induction of IL-4 and IFNγ in plasma was also comparable in both genotypes indicating that Serpinb1a is not essential for this activation pathway in dendritic cells and iNKT cells.

Although Serpinb1a appears as a strong marker for iNKT17 and MAIT17 cells, we have shown that Serpinb1a is dispensable for iNKT cell development and differentiation into the iNKT17 subset. We have also shown that Serpinb1a does not enhance nor impair dendritic cell-mediated activation of cytokine production by iNKT cells following stimulation with αGalCer. iNKT cells have a broad range of actions and act differently depending on the tissue or the context of infection. It remains possible that, in a more complex activation setting with multiple stimuli and/or pathogens, Serpinb1a contributes to altered inflammatory responses in iNKT cells but this remains to be shown. In conclusion, Serpinb1a is not essential for homeostasis and αGalCer-induced responses of iNKT cells and particularly those of iNKT17 cells.

## Data Availability Statement

The raw data supporting the conclusions of this article will be made available by the authors, without undue reservation.

## Ethics Statement

Animal experimentation was conducted in compliance with the Swiss Animal Welfare legislation and animal studies were reviewed and approved by the commission on animal experiments of the canton of Bern, Switzerland under licenses BE8/16 and BE35/19.

## Author Contributions

NL designed and performed experiments, analyzed data, and drafted the manuscript. AT performed experiments. SF provided key technical and scientific advice. CB supervised the project, analyzed data, and wrote the manuscript. All authors contributed to the article and approved the submitted version.

## Funding

This project was funded by grants from the Swiss National Science Foundation (310030-173137) and the Novartis foundation for medical-biological research.

## Conflict of Interest

The authors declare that the research was conducted in the absence of any commercial or financial relationships that could be construed as a potential conflict of interest.
